# Dynamic Covalent Nanoparticle Building Blocks

**DOI:** 10.1002/chem.201601394

**Published:** 2016-06-17

**Authors:** Euan R. Kay

**Affiliations:** ^1^ EaStCHEM School of ChemistryUniversity of St Andrews, North HaughSt Andrews, FifeKY16 9STUK

**Keywords:** dynamic covalent chemistry, nanoparticles, nanostructures, supramolecular chemistry, surface chemistry

## Abstract

Rational and generalisable methods for engineering surface functionality will be crucial to realising the technological potential of nanomaterials. Nanoparticle‐bound dynamic covalent exchange combines the error‐correcting and environment‐responsive features of equilibrium processes with the stability, structural precision, and vast diversity of covalent chemistry, defining a new and powerful approach for manipulating structure, function and properties at nanomaterial surfaces. Dynamic covalent nanoparticle (DCNP) building blocks thus present a whole host of possibilities for constructing adaptive systems, devices and materials that incorporate both nanoscale and molecular functional components. At the same time, DCNPs have the potential to reveal fundamental insights regarding dynamic and complex chemical systems confined to nanoscale interfaces.

## The Importance of Nanoparticle Surface Functionality

Tremendous synthetic and analytical advances over more than two decades have opened up a new region of chemical space on the nanoscale, leading to excitement on account of the extraordinary properties observed for myriad types of nanoparticles (NPs) and other materials in this size regime.^1^ Virtually any potential application of nanomaterials will require careful control over a wide range of features, as well as integration with any number of other components.1d,1e, 2 However, strategies for functionalising and assembling these new chemical entities have not kept pace with advances in their synthesis and there is now a pressing need to bridge this capability gap if the technological potential of nanomaterials is to be realised.

**Figure 1:**
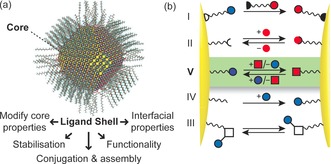
a) Calculated structure of a monolayer‐stabilised NP (5 nm PbS core capped with oleic acid ligands) and the crucial features that are determined by the ligand shell; b) cartoon representation of strategies for postsynthetic NP surface engineering: I. Ligand exchange; II. host–guest complexation; III. stimuli‐responsive molecular switches; IV. kinetically controlled covalent reactions; V. dynamic covalent reactions (highlighted). Panel (a) adapted from ref. 8 with permission from AAAS.

The chemical nature of nanoscale surfaces is inherently critical to the behaviour of all nanomaterials. The canonical example is a monolayer‐stabilised nanoparticle (NP), in which an inorganic core is stabilised by a surface‐bound layer of molecular ligands. The properties of these multicomponent systems are defined both by the material, size and shape of the inorganic core and by the characteristics of the surface‐bound species (Figure 1 a).^3^ Furthermore, the ligand shell provides a handle for attaching other components—be they molecules, surfaces or other nanomaterials. Despite a long history,^4^ the controlled synthesis of NPs of any sort remains challenging and the range of compatible surface functionalities restricted.^5^ ‘Ligand exchange’, whereby temporary surface‐bound molecules are replaced in their entirety (Figure 1 b, I) has been widely exploited for some systems,^6^ yet still presents several practical challenges, has limited scope for linking to complex functionalities or non‐molecular components, and is not applicable to all core materials. Consequently, robust and predictable methods for modifying NP‐bound surface species in a postsynthetic fashion will be critical for manipulating NPs, tuning their properties, and assembling devices and materials from these nanoscale building blocks.^7^ Ideally these methodologies should be independent of the underlying nanomaterial and therefore generalisable across a wide array of nanostructures that can have molecules attached to the surface.

The current strategies for in situ modification of NP‐bound molecules (Figure 1 b, II–IV) have considerable drawbacks that prevent them offering universal solutions. Exploiting noncovalent interactions between biomolecules—and, in particular, hybridisation to single‐stranded oligonucleotides—has proven particularly successful, leading to myriad NP‐based devices and materials.^9^ However, the structural and chemical stability of biomolecules is only maintained within tightly defined conditions; complex high molecular weight architectures limit the scope for structural and chemical variation; and molecular‐level characterisation can be extremely challenging. Nonbiomolecular approaches have the potential to draw upon the full diversity of synthetic chemistry for optimising structure, function and properties.^7^ Innovative designs that exploit noncovalent interactions for NP functionalisation,^10^ aggregation,^11^ and surface immobilisation,^12^ have recently been explored, but still cannot match the stability, specificity, and selectivity of DNA. Stimuli‐responsive molecular switches incorporated within NP ligand shells have recently produced a number of impressive dynamic NP systems, in particular for control of NP aggregation.^13^ The challenges for this relatively unexplored strategy include avoiding degradation processes that lead to switch fatigue, and extending the switching phenomena to properties beyond the aggregation state.^14^

Covalent modification of NP surface functionality is an obvious attractive alternative and has indeed been intensively explored. However, typical examples rely on kinetically controlled reactions, often inspired by mild, robust and high‐yielding protocols developed for bioconjugation applications.^15^ These only offer one‐shot transformations and fail to match the programmability of oligonucleotide approaches. Dynamic covalent bonds—which, under appropriate conditions, can form and break many times over, while under alternative conditions can be kinetically inert—offer a unique solution to these issues (Figure 1 b, V), and the concept of dynamic covalent nanoparticle (DCNP) building blocks opens up a host of possibilities for repeatedly switching—and subtly tuning—NP functionalisation and properties, and for controlling NP self‐assembly.

## Dynamic Covalent Chemistry: Towards Adaptive Chemical Systems, Materials and Surfaces

Processes in which covalent bonds are formed reversibly under conditions of thermodynamic control have for decades been recognised as important in certain branches of chemistry, including carbohydrate stereochemistry^16^ and polymer synthesis.^17^ Inspired by the prospect of extending the newly established principles of supramolecular chemistry into the molecular world—and enabled by advances in analytical technology that made feasible the characterisation of mixtures—in the late 1990s, pioneering groups developed dynamic covalent chemistry as a means of combining the strength, directionality and potential for kinetic stability of covalent bonds, with the benefits of error‐correcting self‐assembly, product stability control, and stimuli‐responsiveness.^18^ Creating equilibrating mixtures of molecular species that could adapt in response to molecular recognition interactions or environmental changes afforded a new strategy for template‐induced selection of optimised supramolecular hosts, guests and catalysts, or for high‐yield assembly of complex molecular architectures.18e, 19 The intervening years have seen rapid progress, leading to several remarkable achievements, including selection of unforeseen macrocyclic receptors from dynamic combinatorial libraries,^20^ construction of three‐dimensional molecular cages and capsules,^21^ preparation of hitherto inaccessible interlocked molecular architectures,^22^ crystallisation of infinite covalent organic frameworks,^23^ self‐replication from a dynamic mixture of competing molecules,^24^ and operation of sophisticated artificial molecular nanomachines.^25^ Logical extensions of these concepts have led to the self‐assembly of responsive and adaptive materials,^26^ including dynamic covalent polymers^27^ and self‐selecting small molecule ‘dynablocks’.^28^

Characterisation of inherently dynamic and multicomponent chemical systems is a formidable challenge in any setting,^29^ even more so when moving away from the familiar solution‐state environment into condensed phases or on to surfaces.19b Dynamic covalent processes occurring at interfaces have been correspondingly demanding to establish. Pioneering studies exploited emerging characterisation techniques including atomic force microscopy and confocal fluorescence microscopy to visualise erasable molecular patterns^30^ and directed surface diffusion of covalently attached macromolecules,^31^ operating by reversible condensation and hydrolysis of imines on self‐assembled monolayers (SAMs). More recently, pH‐controlled patterning of imine SAMs was achieved through dynamic covalent selection from a mixture of small‐molecule amines or amine‐containing proteins.^32^ Multiple orthogonal dynamic covalent processes have since been combined to construct an impressive series of chemically responsive multicomponent surface architectures in which several coaxially aligned photoconductive channels can be grown normal to the surface with control over patterning parallel to the substrate.^33^

Enabled by developments in scanning probe microscopy, potentially reversible covalent reactions have also been investigated for the construction of extended noncovalent and covalent atomically thin 2D networks at solid surfaces under vacuum,^34^ at the solid/liquid interface^35^ and at the liquid/air interface.^36^ Recently, dynamic covalent exchange was demonstrated in one such system, where the free energy of surface physisorption can act as a driving force for constituent selection and amplification from a dynamic library of imines.^37^

The emergence of surface‐confined dynamic covalent systems now suggests several compelling possibilities, including spatiotemporal control over the exchange process, leading to the prospect of creating surface patterns or achieving compartmentalised chemical behaviour spanning several length scales. These studies have helped to reveal the often considerable influence of the unique surface microenvironment when chemical processes are confined to interfaces.

## Dynamic Covalent Nanoparticle Building Blocks

These contemporaneous developments for both nanomaterials and dynamic molecular systems now set the stage for extending dynamic covalent reactions onto nanosurfaces. Intermediate in size between macromolecules and extended surfaces, applying reversible covalent reactions to nanosurfaces allows the principles of equilibrium control to be applied to achieve responsive and adaptive behaviour on the nanoscale, while exploiting the precision and diversity of synthetic molecular structures. Working with nanosized chemical entities introduces several new challenges, but also opens up exciting possibilities for elucidating fundamental features of chemical processes taking place at interfaces that are just not available for extended substrates.

In a handful of cases, cargoes have been attached to and then released from nanomaterials through formation and cleavage of the same covalent bond. For example, inspired by stationary phases developed for affinity chromatography, boronic acids have been associated with magnetic NPs and used to isolate and enrich polyhydroxylated biomolecules from biological samples followed by release for analysis by mass spectrometry.^38^ Boronic acid functionalised gold nanoparticles (AuNPs) have also been used to reversibly trap molecular cargoes in the pores of silica solids that display polyhydroxyl surface functionality; cargo release being achieved by lowering pH, consistent with a mechanism involving pH‐switched boronate ester condensation/hydrolysis.^39^ A similar approach has been used for the reversible gating of mesoporous silica nanoparticles (MSNPs), where the silica surface was functionalised with boronic acids and the capping unit was the polyhydroxylated glycoprotein insulin.^40^ Moreover, imine linkages have been used to attach and detach hydrophobic dendrons on the surface of silica‐coated superparamagnetic microspheres^41^ and both hydrazones^42^ and oximes^43^ have been used for loading and release of simple carbonyl‐containing units on MSNPs.

Taking place on sparsely‐functionalised irregular surfaces, direct characterisation of the molecular processes in such systems is a formidable challenge. These examples all exploit efficient interconversion of functional groups through high‐yielding condensation and hydrolysis reactions. Under each set of conditions, formation or cleavage of a covalent bond is essentially irreversible and is therefore conceptually similar to other covalent surface modifications.^15^ However, dynamic covalent exchange processes would allow subtle and responsive tuning of the NP surface functionality, controlled by thermodynamic differences between a number of exchanging species, and exhibiting constitutional adaptation in response to myriad parameters and interactions. Dynamic covalent nanoparticle (DCNP) building blocks therefore introduce a powerful new approach to nanomaterial surface engineering, and the construction of responsive NP‐based devices, assemblies and materials.

### Dynamic covalent exchange on phospholipid nanosurfaces

**Figure 2:**
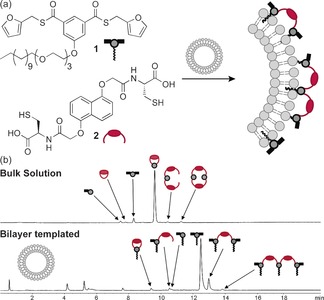
a) Schematic representation of a bilayer‐templated dynamic combinatorial library formed by thioester exchange between surface‐active bis‐thioester **1** and dithiol **2** in the presence of lipid bilayer vesicles; b) HPLC traces comparing library compositions produced in bulk aqueous solution (top trace) and at the lipid bilayer interface (bottom trace, 10 mm egg phosphatidyl choline, 1.0 mm **1** and 1.0 mm **2**).^45^ Adapted from ref. 45 under the terms of CC‐BY 2.0.

Myriad dynamic biomolecular reaction and recognition processes take place on phospholipid bilayers; for example, disulfide exchange with exofacial thiols on membrane‐associated proteins can mediate signalling pathways, viral entry and non‐endosomal cellular uptake processes.^44^ In addition to the considerable analytical challenges, achieving dynamic covalent reactions on artificial nanoscale membranes must also overcome the often limited stability of self‐assembled amphiphile structures. Nevertheless, Otto and co‐workers successfully demonstrated dynamic covalent thioester exchange on the surface of large (*d*≈200 nm) unilamellar vesicles assembled from egg phosphatidyl choline and 10 mol % of a membrane‐anchored thioester derivative **1** (Figure 2).^45^ Surface confinement was found to retard the dynamic covalent thioester exchange reactions only to a modest extent and equilibrium control was maintained. Significantly, thioester libraries equilibrated on these liposome surfaces exhibited markedly different compositions compared to bulk solution. For example, when amphiphilic bis‐thioester **1** was equilibrated with dithiol **2**, a high proportion of linear compounds (formed by cross‐linking several membrane‐anchored units) was observed, in contrast to the small monomeric and dimeric products formed in solution (Figure 2 b). This can be attributed to the higher local concentration of **1** at the interface, compared to when at the same overall concentration in bulk solution.

### Dynamic covalent exchange on monolayer‐stabilised inorganic nanosurfaces

**Figure 3:**
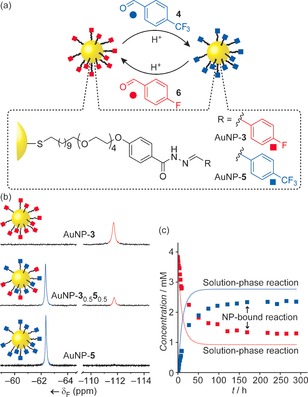
a) Dynamically reconfigurable AuNP‐bound hydrazone monolayers; b) in situ characterisation by ^19^F NMR spectroscopy allows molecular‐level characterisation of monolayer composition; c) real‐time tracking of dynamic covalent exchange reactions.^46^ Adapted with permission from ref. 46, copyright © Wiley‐VCH Verlag GmbH & Co. KGa, Weinheim.

Our group first explored the DCNP concept by using AuNPs (*d*≈3.0 nm) bearing a hydrazone‐terminated surface monolayer (AuNP‐**3**; Figure 3).^46^ On introducing an aldehyde exchange unit, such as **4**, together with an acid catalyst, the monolayer composition could be reliably and reversibly modified through dynamic covalent hydrazone exchange. The single‐component monolayer achieves extremely high surface densities of exchangeable units, which could be quantitatively modified; or alternatively, mixed monolayers could be accessed with compositions defined by the thermodynamic stability of each hydrazone and the reaction conditions. Simply removing the acid catalyst afforded kinetically stable products that could be isolated, purified, characterised, and stored without further changes to the monolayer.

In contrast to larger and sparsely functionalised nanosurfaces—which behave in many respects as curved analogues of 2D extended SAMs—these small colloidal NPs, stabilised by a dense, single‐component monolayer, are perhaps better considered as a unique category of 3D SAMs, with many features akin to large macromolecules.^47^ This pseudomolecular nature allows analytical tools such as NMR spectroscopy to be exploited for in situ characterisation of surface‐bound structures and processes; opportunities that are not applicable to analogous 2D systems or sparsely functionalised larger NPs. Monitoring DCNP hydrazone exchange by ^19^F NMR spectroscopy allowed both NP‐bound and unbound species to be quantified in real time (Figure 3 b,c). As might be intuitively expected, the surface‐confined process proceeded more slowly than analogous solution‐phase reactions.^46^ However, the retardation was relatively modest and dynamic covalent exchange was found to be significantly faster than analogous ligand exchange processes at the Au−S bond, which of course also first require each new ligand to be synthetically prepared. The ability to characterise molecular‐level details promises a quantitative understanding of NP‐bound dynamic covalent reactions that will underpin rational synthetic methods for manipulating DCNPs with predictability and precision.

**Figure 4:**
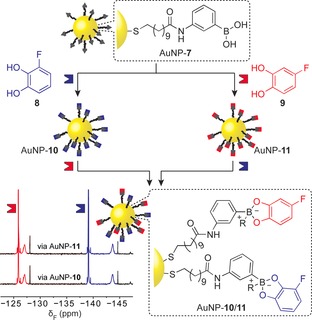
NP‐bound boronate ester formation and dynamic covalent exchange on sequential reaction of AuNP‐**7** with catechols **8** (blue) and **9** (red). In situ analysis by ^19^F NMR spectroscopy reveals identical mixed monolayer equilibria are reached via two different routes (R=*N*‐methylmorpholinium).^48^ Adapted from ref. 48 with permission from The Royal Society of Chemistry.

One of the advantages of the DCNP concept is the ability to exploit the vast toolbox of synthetic organic chemistry to tune virtually any aspect of the surface‐bound molecular structure, properties or reactivity. Whereas hydrazone‐exchange reactions tend to reach equilibrium on a timescale of minutes to hours under acid‐ or nucleophile‐catalysed conditions, the reaction between a boronic acid and various dihydroxy compounds to yield boronate esters occurs extremely rapidly in the presence of Lewis bases. Hydrazones and boronate esters therefore represent two chemically orthogonal dynamic covalent functional groups with an attractive contrast in kinetic characteristics. We recently developed AuNP‐**7** (*d*≈3.4 nm; Figure 4), stabilised by a homogenous monolayer of structurally simple boronic acids, which reacts with 1,2‐diols in the presence of a Lewis base to provide NP‐bound boronate esters.^48^ In situ analysis by ^19^F NMR spectroscopy revealed that boronate ester formation and dynamic covalent exchange (when more than one diol‐containing exchange unit is introduced) occurs rapidly and reversibly, achieving high surface‐saturation concentrations. Mixed boronate ester monolayers display pathway‐independent compositions that adapt to changes in the mixture of exogenously introduced diols, confirming that dynamic covalent exchange on the NP surface remains an equilibrium process under thermodynamic control (Figure 4).^48^

**Figure 5:**
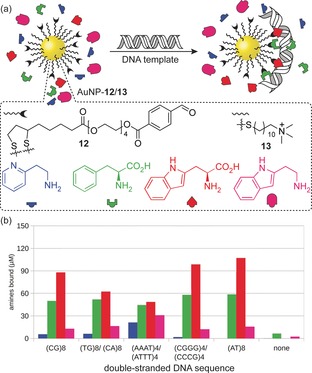
a) DNA‐templated dynamic covalent AuNP‐bound imine monolayers; negligible imine formation occurs in the absence of DNA; b) monolayer composition depends on the specific base pair sequence of the double‐stranded DNA template.^49^ Panel (b) adapted with permission from ref. 49, copyright © Wiley‐VCH Verlag GmbH & Co. KGa, Weinheim.

Independently, Otto and co‐workers have exploited kinetically facile imine exchange to achieve template‐driven dynamic covalent NP functionalisation (Figure 5).^49^ On treating AuNP‐**12**/**13** (*d*≈11.7 nm), which is stabilised by a mixed monolayer incorporating approximately 10 mol % aldehyde ligand **12**, with mixtures of simple primary amines, negligible NP‐bound imine formation was observed. However, on introducing short (16‐mer) oligonucleotides, selective uptake of amines from solution was observed. This could be explained by thermodynamic stabilisation of NP‐bound imines as a result of multivalent interactions between the imine‐functionalised NP surface and the oligonucleotide template. Significantly, the degree of functionalisation, and the composition of NP‐bound imine libraries, depended on the oligonucleotide sequence employed (Figure 5 b): the dynamic covalent ligand shell can adapt to optimise binding to each specific DNA template.^49^ Very recently, the same approach was extended to the formation of NP‐bound hydrazones, whereby mixtures of two different NP‐bound hydrazones were obtained depending on the DNA template sequence.^50^ This second‐generation system also has the advantage of employing a neutral surface monolayer, which minimises nonspecific binding to the anionic oligonucleotide templates.

Together, these studies establish that dynamic molecular exchange chemistry can be used to selectively control NP functionalisation through adaptive surface monolayers, as well as pointing to the potential insights—and considerable challenges—associated with characterising dynamic covalent processes in this unconventional environment. The diversity of abiotic synthetic chemistry may now be exploited to develop a whole series of DCNP building blocks that would constitute a universal set of environment‐responsive ‘nanoparticle synthons’ with optimised reactivity and properties for a wide range of applications.

## Reversible Control of DCNP Properties

**Figure 6:**
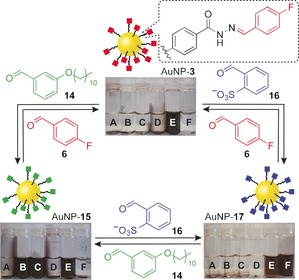
Reversible control of DCNP solvent compatibility by dynamic covalent hydrazone exchange; solvents: A=hexane, B=chloroform, C=tetrahydrofuran, D=methanol, E=*N*,*N*‐dimethylformamide, F=water.^46^ Adapted with permission from ref. 46, copyright © Wiley‐VCH Verlag GmbH & Co. KGa, Weinheim.

Mild methods for postsynthetic NP property control are highly desirable and would have significant benefits for nanomaterial handling and processing. We reported how hydrazone exchange can be used to achieve reversible and stimuli‐responsive control over DCNP physicochemical properties by appropriate choice of molecular exchange unit (Figure 6).^46^ AuNP‐**3** was colloidally stable only in polar aprotic solvents, yet hydrazone exchange with hydrophobic aldehyde **14** produced AuNP‐**15**, which exhibited colloidal stability in less polar organic solvents. Likewise, exchange with charged aldehyde **16** produced AuNP‐**17**, which was colloidally stable in water. In contrast to alternative approaches such as ligand exchange or noncovalent encapsulation, DCNPs combine excellent stability in each state with complete reversibility; any one state can be accessed from any of the others, and each state corresponds to a single, covalently bound entity, with no requirement for weakly associated stabilisers or surfactants.

**Figure 7:**
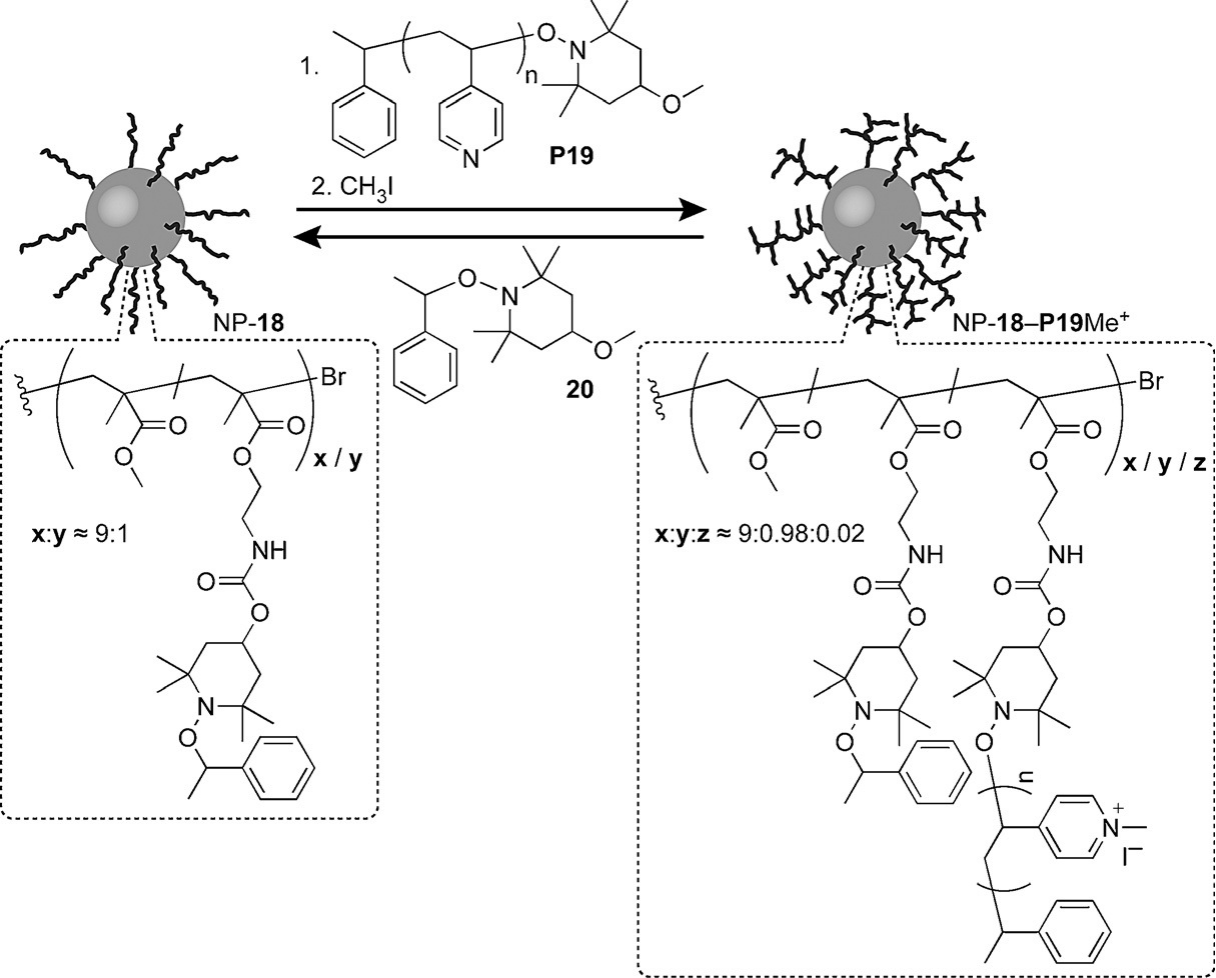
Dynamic covalent polymer brush‐functionalised silica NPs. Alkoxyamine exchange can be used to reversibly attach macromolecular poly(4‐vinylpyridine) side chains **P19**. Subsequent quaternisation of pyridine nitrogen atoms results in a hydrophilic NP coating that can subsequently be removed by dynamic covalent exchange with small molecule **20**.^51^

In a recent study, Takahara, Otsuka and co‐workers prepared large silica NPs (*d*≈100 nm) functionalised with polymer brushes bearing approximately 10 mol % alkoxyamine side chains (NP‐**18**, Figure 7).^51^ The dynamic covalent radical crossover reaction of alkoxyamines was then exploited to graft on appropriately functionalised polymer exchange units, such as poly(4‐vinylpyridine) derivative **P19**. Although the extent of exchange was very limited (ca. 2.2 % as quantified by XPS analysis), subsequent quaternisation of the pyridine units rendered silica NP‐**18**−**P19Me^+^** dispersible in water. Reaction with the small‐molecule alkoxyamine **20** recovered both the XPS signature and solvent compatibility properties of the initial sample, consistent with a fully reversible dynamic covalent exchange process.^51^

## Molecule‐Directed Assembly of DCNP Building Blocks

Structural control over nanoparticle assemblies is important for defining a number of emergent and collective properties when several NPs are brought together, and is therefore of crucial importance to many innovative NP applications, from sensing to catalysis; optoelectronics to thermoelectrics.1d,1e Furthermore, integrating nanostructures with components from existing technologies, such as microelectronics or optics, requires the controlled assembly and patterning of NPs across several size scales.^2^ Although NP ‘superlattices’ may be assembled, driven by nonspecific dispersion forces on controlled solvent evaporation, these tend to form close‐packed structures with arrangements that are specified by the size and shape of the NP building blocks.^52^ Independent control over NP building block characteristics and assembly structure through chemistry‐led design of interparticle linkers has been exemplified by the emergence of ordered, non‐close‐packed NP arrays connected by oligonucleotide hybridisation.9g However, all of the attendant issues with regards to DNA stability and customisability remain, and assembly at surfaces is still very much in its infancy.^53^ Despite several notable advances exploiting nonbiomolecular linkers,^11^, ^54^ all‐covalent strategies tend to encounter kinetic traps, whereas noncovalent approaches can require very specific conditions or complex molecular designs to produce well‐defined kinetically stable structures.

**Figure 8:**
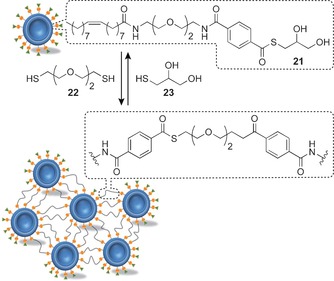
Reversible liposome cross‐linking by dynamic covalent thioester exchange.^55^ Adapted from ref. 55 with permission from The Royal Society of Chemistry.

DCNP building blocks have the exciting potential to self‐assemble under error‐correcting, thermodynamically controlled conditions, yet be connected by structurally unambiguous, stable and diverse covalent linkers. Exploiting dynamic covalent exchange of bilayer‐confined thioesters, Ravoo and co‐workers reported the reversible assembly of liposome aggregates.^55^ Liposomes (*d*≈100 nm) constructed from soy bean lecithin and 25 mol % thioester **21** were treated with dithiol **22**, resulting in covalent cross‐links between the vesicles (Figure 8). Although the assembly morphology has not been determined, dynamic light scattering indicated the formation of polydisperse aggregates over a period of several hours, which could subsequently be disrupted by addition of an excess of monofunctional thiol **23** to drive the dynamic covalent exchange process back towards the starting thioester.

An optical sensor for assessing enantiomeric excess of chiral diols has been demonstrated, based on enantioselective disruption of colloidally stable aggregates produced from saccharide‐functionalised AuNPs in the presence of inorganic borate anions.^56^ Aggregation, which produces an easily detectible optical signature on account of changes to the AuNP‐localised surface plasmon resonance, was attributed to the formation of dynamic covalent spiroborate cross‐links between NP‐bound vicinal diols. These linkages are subsequently disrupted on introduction of the small‐molecule diol analytes.

**Figure 9:**
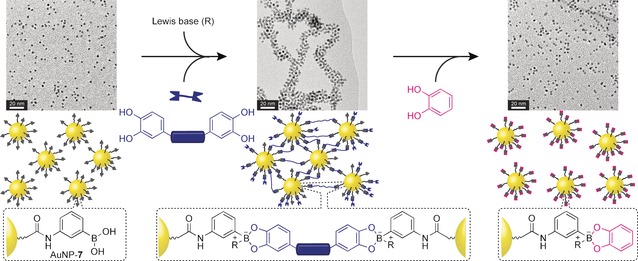
Boronate ester‐driven DCNP assembly and disassembly on sequential addition of a bifunctional linker (blue), followed by a monofunctional capping unit (pink).^48^ Adapted from ref. 48 with permission from The Royal Society of Chemistry.

Combining organic molecule linkers with monolayer‐stabilised DCNPs, we recently demonstrated the dynamic covalent assembly of extended and responsive NP aggregates by using boronic acid‐functionalised AuNP‐**7** (Figure 4 and 9) in combination with ditopic linkers that can themselves be varied to tune the aggregate morphology in a modular fashion.^48^ In the presence of a bifunctional diol linker, dynamic covalent boronate ester cross‐links are formed between the DCNPs. The resulting extended aggregates eventually precipitate from solution, producing low‐density open networks that are consistent with a diffusion‐limited aggregation process. Assembly is quantitative and switchable: the DNCP building block and molecular linker do not interact until addition of a Lewis base to stabilise the NP‐bound boronate esters, thus initiating assembly. Remarkably, despite being linked by covalent bonds, the resulting solid‐state aggregates can then be completely disassembled by introducing a competitive monofunctional diol (Figure 9).^48^

Interestingly, DCNP aggregate morphology was found to vary depending on the nature of the bifunctional linker, suggesting that morphology can be tuned through structural modification of a small‐molecule component. With the ability to characterise NP‐bound dynamic covalent processes in situ (vide supra), this offers a straightforward and rational approach to varying morphological parameters over a wider range than is possible using biomolecular systems. This modular strategy might also be extended to incorporate additional chemical, physical or structural features within the molecular linker design. The diversity of complementary and orthogonal dynamic covalent exchange processes points the way to yet‐more sophisticated assemblies and devices that incorporate several different DCNP building blocks, designed to be selectively reactive with each other and/or with specific species. The DCNP building block concept thus suggests several intriguing avenues towards responsive, reconfigurable and truly multifunctional hybrid nanomaterials in which both molecular and nanoscale components are precisely arranged, and combine to define emergent system properties.

## Conclusion and Outlook

One only has to look to biology to understand the potential for producing remarkable functional materials or complex chemical networks by nanoscale confinement of dynamic molecular systems on interfaces or within compartments. Advances in both synthetic and analytical technology are now allowing chemists to consider emulating some of these extraordinary systems, even if only at a comparatively rudimentary level. Dynamic covalent reactions offer unique advantages in their combination of specific and selective thermodynamically controlled reactivity, within structurally robust and unambiguous small‐molecule covalent structures that are synthetically and analytically tractable. Conferring dynamic covalent reactivity on the vast array of nanomaterials that can now be generated with increasingly precise control over chemical composition, size, shape and dispersity, will therefore establish a versatile new category of nanochemical synthon: the *DCNP building block*.

Whether on soft or hard nanosurfaces, combining the error‐correcting and environment‐responsive features of equilibrium processes with the stability and structural diversity of covalent chemistry amounts to a ‘best of both worlds’ solution to the problem of engineering surface functionality. The basic concept is largely independent of the nanomaterial itself, and so can ultimately be generalised across a range of materials, shapes and sizes. This is in stark contrast to ligand‐exchange strategies, which by definition must be specific for each different nanomaterial–molecule interaction; dynamic covalent exchange of simple units on the periphery of a stabilising monolayer can occur significantly faster under milder conditions and avoids the necessity for multistep synthesis of each ligand from scratch. The advantages of a nonbiomolecular approach include the ability to readily vary structural and chemical parameters, and operate under a wide range of conditions. The ever‐increasing number of well‐characterised dynamic covalent reactions19c, 57 affords a diversity of complementary and orthogonal reactivities, spanning a huge range of kinetic behaviours and operating conditions. Furthermore, with the full gamut of synthetic small‐molecule chemistry at our disposal, the possibilities for augmenting both structure and properties through the supporting molecular scaffold are almost limitless. For all these reasons, DCNPs can provide a toolbox of universal nanoscale building blocks that can be predictably modified, combined, and assembled to suit numerous of applications.

The pseudomolecular nature of colloidally stable NPs—at least at the lower end of the size‐scale—means that although the analytical challenges are significant, they are not insurmountable. Several studies discussed herein have demonstrated that these systems can reveal direct insight into chemical processes occurring at interfaces, something that has proven extremely challenging in other settings. Consequently, DCNPs constitute a unique platform on which to study surface‐bound molecular behaviour. Understanding the subtle and complex network of interactions governing reactivity within NP‐bound monolayers will be crucial to arriving at rational and predictable synthetic methodologies for working with DCNPs, for example by quantifying and understanding the influence of surface confinement on reaction kinetics.^46^ At the same time, such fundamental questions are equally relevant to better understanding a variety of other surface‐confined molecular processes, from heterogeneous catalysis to cell‐surface recognition. The profound influence of the surface‐bound microenvironment on the thermodynamics of equilibrating dynamic covalent systems has already been demonstrated,^45^, ^49^ and points the way towards templated nanosurfaces that array numerous copies of several functionalities across a nanoscale surface area, thus providing synthetic analogues for modelling or perturbing large‐area multivalent interactions that are at the heart of numerous natural processes.^58^

The essential role that surface‐bound species play in defining a range of nanomaterial properties means that dynamic covalent modification heralds a powerful, environment‐responsive route to controlling system‐level behaviours, including physicochemical properties or microscopic and mesoscopic assembly structures. We have shown that this approach can be used to reversibly switch and tune NP solvent compatibility using very simple molecular exchange units,^46^ or else to assemble covalently linked NP aggregates that are responsive to specific chemical stimuli.^48^ There is rich potential for creating adaptive and reconfigurable devices and materials in which structural features and physicochemical properties can be tuned in a modular fashion by rational selection of molecular and NP components that are assembled in thermodynamically controlled error‐correcting processes and may be perturbed by external stimuli.

Mastering complex systems that arise through the interaction of dynamic chemical processes, reaction networks and structures is one of the next grand challenges facing synthetic, analytical and theoretical chemists in the coming decades;^59^ dynamic covalent chemistry and nanomaterial building blocks will undoubtedly play significant roles in realising this. Although the concept of a rational ‘heterosupramolecular chemistry’ whereby supramolecular chemistry principles could be applied to achieve molecular‐level control over nanoparticles was proposed at least two decades ago,^60^ it is only now that synthetic and analytical capabilities have reached a level of maturity that allows such aspirations to be met. Dynamic covalent reactions are being exploited in all sorts of original ways,^20^, ^21^, ^22^, ^23^, ^24^, ^25^, ^26^, ^27^, ^28^, ^29^, ^30^, ^31^, ^32^, ^33^, ^34^, ^35^, ^36^, ^37^ including in systems that are coupled to external energy sources, such as out‐of‐equilibrium chemical reactions,24a transmembrane concentration gradients^61^ or repeatedly applied external stimuli.^62^ We can expect that cross‐fertilisation between each of these themes will serve as inspiration for ever more innovative designs and complex behaviours on the nanoscale. With the ability to reveal fundamental insights, and transformative potential as an enabling technology, DCNPs will contribute to this future chemical synthetic science, whereby molecular, nanoscale and macroscopic building blocks may be combined with equal precision, to create complex, responsive and adaptive systems operating across several size scales.

## References

[1a] G. A. Ozin , A. C. Arsenault , Nanochemistry. A Chemical Approach to Nanomaterials, The Royal Society of Chemistry, Cambridge, 2005;

[1b] C. Burda , X. B. Chen , R. Narayanan , M. A. El-Sayed , Chem. Rev. 2005, 105, 1025–1102; 15826010 10.1021/cr030063a

[1c] H. Goesmann , C. Feldmann , Angew. Chem. Int. Ed. 2010, 49, 1362–1395; 10.1002/anie.200903053 20108287

[1d] D. V. Talapin , J.-S. Lee , M. V. Kovalenko , E. V. Shevchenko , Chem. Rev. 2010, 110, 389–458; 19958036 10.1021/cr900137k

[1e] P. D. Howes , R. Chandrawati , M. M. Stevens , Science 2014, 346, 1247390. 25278614 10.1126/science.1247390

[2a] Z. H. Nie , A. Petukhova , E. Kumacheva , Nat. Nanotechnol. 2010, 5, 15–25; 20032986 10.1038/nnano.2009.453

[2b] L. Xu , W. Ma , L. Wang , C. Xu , H. Kuang , N. A. Kotov , Chem. Soc. Rev. 2013, 42, 3114–3126. 23455957 10.1039/c3cs35460a

[3a] R. A. Sperling , W. J. Parak , Philos. Trans. R. Soc. London Ser. A 2010, 368, 1333–1383; 10.1098/rsta.2009.0273 20156828

[3b] M. A. Boles , D. Ling , T. Hyeon , D. V. Talapin , Nat. Mater. 2016, 15, 141–153. 26796733 10.1038/nmat4526

[4] M. Faraday , Philos. Trans. R. Soc. London 1857, 147, 145–181.

[5a] J. Park , J. Joo , S. G. Kwon , Y. Jang , T. Hyeon , Angew. Chem. Int. Ed. 2007, 46, 4630–4660; 10.1002/anie.200603148 17525914

[5b] Y. Xia , Y. Xiong , B. Lim , S. E. Skrabalak , Angew. Chem. Int. Ed. 2009, 48, 60–103; 10.1002/anie.200802248 PMC2791829 19053095

[5c] N. T. K. Thanh , N. Maclean , S. Mahiddine , Chem. Rev. 2014, 114, 7610–7630. 25003956 10.1021/cr400544s

[6a] M. J. Hostetler , S. J. Green , J. J. Stokes , R. W. Murray , J. Am. Chem. Soc. 1996, 118, 4212–4213;

[6b] R. S. Ingram , M. J. Hostetler , R. W. Murray , J. Am. Chem. Soc. 1997, 119, 9175–9178;

[6c] A. Caragheorgheopol , V. Chechik , Phys. Chem. Chem. Phys. 2008, 10, 5029–5041. 18701949 10.1039/b805551c

[7] W. Edwards , E. R. Kay , ChemNanoMat 2016, 2, 87–98.

[8] D. Zherebetskyy , M. Scheele , Y. Zhang , N. Bronstein , C. Thompson , D. Britt , M. Salmeron , P. Alivisatos , L.-W. Wang , Science 2014, 344, 1380–1384. 24876347 10.1126/science.1252727

[9a] E. Katz , I. Willner , Angew. Chem. Int. Ed. 2004, 43, 6042–6108; 10.1002/anie.200400651 15538757

[9b] S. Mann , W. Shenton , M. Li , S. Connolly , D. Fitzmaurice , Adv. Mater. 2000, 12, 147–150;

[9c] S. S. Behrens , J. Mater. Chem. 2008, 18, 3788–3798;

[9d] C. L. Choi , A. P. Alivisatos , Annu. Rev. Phys. Chem. 2010, 61, 369–389; 20055683 10.1146/annurev.physchem.012809.103311

[9e] C.-L. Chen , N. L. Rosi , Angew. Chem. Int. Ed. 2010, 49, 1924–1942; 10.1002/anie.200903572 20183835

[9f] J. I. Cutler , E. Auyeung , C. A. Mirkin , J. Am. Chem. Soc. 2012, 134, 1376–1391; 22229439 10.1021/ja209351u

[9g] R. J. Macfarlane , M. N. O'Brien , S. H. Petrosko , C. A. Mirkin , Angew. Chem. Int. Ed. 2013, 52, 5688–5698; 10.1002/anie.201209336 23640804

[10a] U. Drechsler , B. Erdogan , V. M. Rotello , Chem. Eur. J. 2004, 10, 5570–5579; 15372582 10.1002/chem.200306076

[10b] V. Montes-García , J. Pérez-Juste , I. Pastoriza-Santos , L. M. Liz-Marzán , Chem. Eur. J. 2014, 20, 10874–10883; 25043786 10.1002/chem.201403107

[10c] Y.-W. Yang , Y.-L. Sun , N. Song , Acc. Chem. Res. 2014, 47, 1950–1960; 24635353 10.1021/ar500022f

[10d] L. J. Prins , Acc. Chem. Res. 2015, 48, 1920–1928. 26098550 10.1021/acs.accounts.5b00173

[11a] A. K. Boal , F. Ilhan , J. E. DeRouchey , T. Thurn-Albrecht , T. P. Russell , V. M. Rotello , Nature 2000, 404, 746–748; 10783884 10.1038/35008037

[11b] R. Klajn , M. A. Olson , P. J. Wesson , L. Fang , A. Coskun , A. Trabolsi , S. Soh , J. F. Stoddart , B. A. Grzybowski , Nat. Chem. 2009, 1, 733–738; 21124361 10.1038/nchem.432

[11c] T. Shirman , T. Arad , M. E. van der Boom , Angew. Chem. Int. Ed. 2010, 49, 926–929; 10.1002/anie.200905984 20029869

[11d] J. Zhang , R. J. Coulston , S. T. Jones , J. Geng , O. A. Scherman , C. Abell , Science 2012, 335, 690–694. 22323815 10.1126/science.1215416

[12a] D. Dorokhin , S.-H. Hsu , N. Tomczak , D. N. Reinhoudt , J. Huskens , A. H. Velders , G. J. Vancso , ACS Nano 2010, 4, 137–142; 20020751 10.1021/nn901109x

[12b] T. Shirman , R. Kaminker , D. Freeman , M. E. van der Boom , ACS Nano 2011, 5, 6553–6563. 21749110 10.1021/nn201923q

[13a] R. Klajn , J. F. Stoddart , B. A. Grzybowski , Chem. Soc. Rev. 2010, 39, 2203–2237; 20407689 10.1039/b920377j

[13b] A. C. Fahrenbach , S. C. Warren , J. T. Incorvati , A.-J. Avestro , J. C. Barnes , J. F. Stoddart , B. A. Grzybowski , Adv. Mater. 2013, 25, 331–348; 22933356 10.1002/adma.201201912

[13c] H. Zhao , S. Sen , T. Udayabhaskararao , M. Sawczyk , K. Kučanda , D. Manna , P. K. Kundu , J.-W. Lee , P. Král , R. Klajn , Nat. Nanotechnol. 2015, 11, 82–88. 26595335 10.1038/nnano.2015.256

[14] E. R. Kay , J. Lee , D. G. Nocera , M. G. Bawendi , Angew. Chem. Int. Ed. 2013, 52, 1165–1169; 10.1002/anie.201207181 PMC3793206 23225635

[15] K. E. Sapsford , W. R. Algar , L. Berti , K. B. Gemmill , B. J. Casey , E. Oh , M. H. Stewart , I. L. Medintz , Chem. Rev. 2013, 113, 1904–2074. 23432378 10.1021/cr300143v

[16] E. L. Eliel , S. H. Wilen , Stereochemistry of Organic Compounds, Wiley-Interscience, New York, 1994.

[17] F. S. Dainton , K. J. Ivin , Nature 1948, 162, 705–707.

[18a] P. A. Brady , J. K. M. Sanders , Chem. Soc. Rev. 1997, 26, 327–336;

[18b] J. M. Lehn , Chem. Eur. J. 1999, 5, 2455–2463;

[18c] G. R. L. Cousins , S.-A. Poulsen , J. K. M. Sanders , Curr. Opin. Chem. Biol. 2000, 4, 270–279; 10826973 10.1016/s1367-5931(00)00088-0

[18d] J. M. Lehn , A. V. Eliseev , Science 2001, 291, 2331–2332; 11269307 10.1126/science.1060066

[18e] S. J. Rowan , S. J. Cantrill , G. R. L. Cousins , J. K. M. Sanders , J. F. Stoddart , Angew. Chem. Int. Ed. 2002, 41, 898–952; 10.1002/1521-3773(20020315)41:6<898::aid-anie898>3.0.co;2-e 12491278

[19a] P. T. Corbett , J. Leclaire , L. Vial , K. R. West , J.-L. Wietor , J. K. M. Sanders , S. Otto , Chem. Rev. 2006, 106, 3652–3711; 16967917 10.1021/cr020452p

[19b] J. Li , P. Nowak , S. Otto , J. Am. Chem. Soc. 2013, 135, 9222–9239; 23731408 10.1021/ja402586c

[19c] Y. Jin , C. Yu , R. J. Denman , W. Zhang , Chem. Soc. Rev. 2013, 42, 6634–6654. 23749182 10.1039/c3cs60044k

[20] S. Otto , R. L. E. Furlan , J. K. M. Sanders , Science 2002, 297, 590–593. 12142534 10.1126/science.1072361

[21] P. Mal , B. Breiner , K. Rissanen , J. R. Nitschke , Science 2009, 324, 1697–1699. 19556504 10.1126/science.1175313

[22a] K. S. Chichak , S. J. Cantrill , A. R. Pease , S.-H. Chiu , G. W. V. Cave , J. L. Atwood , J. F. Stoddart , Science 2004, 304, 1308–1312; 15166376 10.1126/science.1096914

[22b] D. A. Leigh , R. G. Pritchard , A. J. Stephens , Nat. Chem. 2014, 6, 978–982. 25343602 10.1038/nchem.2056

[23a] H. M. El-Kaderi , J. R. Hunt , J. L. Mendoza-Cortés , A. P. Côté , R. E. Taylor , M. O'Keeffe , O. M. Yaghi , Science 2007, 316, 268–272; 17431178 10.1126/science.1139915

[23b] J. W. Colson , A. R. Woll , A. Mukherjee , M. P. Levendorf , E. L. Spitler , V. B. Shields , M. G. Spencer , J. Park , W. R. Dichtel , Science 2011, 332, 228–231; 21474758 10.1126/science.1202747

[23c] Y. Liu , Y. Ma , Y. Zhao , X. Sun , F. Gándara , H. Furukawa , Z. Liu , H. Zhu , C. Zhu , K. Suenaga , P. Oleynikov , A. S. Alshammari , X. Zhang , O. Terasaki , O. M. Yaghi , Science 2016, 351, 365–369. 26798010 10.1126/science.aad4011

[24a] J. W. Sadownik , D. Philp , Angew. Chem. Int. Ed. 2008, 47, 9965–9970; 10.1002/anie.200804223 19006137

[24b] R. Nguyen , L. Allouche , E. Buhler , N. Giuseppone , Angew. Chem. Int. Ed. 2009, 48, 1093–1096; 10.1002/anie.200804602 19115349

[24c] J. M. A. Carnall , C. A. Waudby , A. M. Belenguer , M. C. A. Stuart , J. J.-P. Peyralans , S. Otto , Science 2010, 327, 1502–1506. 20299594 10.1126/science.1182767

[25a] M. von Delius , E. M. Geertsema , D. A. Leigh , Nat. Chem. 2010, 2, 96–101; 21124398 10.1038/nchem.481

[25b] S. Kassem , A. T. L. Lee , D. A. Leigh , A. Markevicius , J. Solà , Nat. Chem. 2016, 8, 138–143. 26791896 10.1038/nchem.2410

[26] E. Moulin , G. Cormos , N. Giuseppone , Chem. Soc. Rev. 2012, 41, 1031–1049. 21909573 10.1039/c1cs15185a

[27a] J. M. Lehn , Prog. Polym. Sci. 2005, 30, 814–831;

[27b] T. Maeda , H. Otsuka , A. Takahara , Prog. Polym. Sci. 2009, 34, 581–604;

[27c] R. J. Wojtecki , M. A. Meador , S. J. Rowan , Nat. Mater. 2011, 10, 14–27; 21157495 10.1038/nmat2891

[27d] C. J. Kloxin , C. N. Bowman , Chem. Soc. Rev. 2013, 42, 7161–7173; 23579959 10.1039/c3cs60046g

[27e] C. S. Mahon , D. A. Fulton , Nat. Chem. 2014, 6, 665–672. 25054935 10.1038/nchem.1994

[28] N. Giuseppone , Acc. Chem. Res. 2012, 45, 2178–2188. 22533472 10.1021/ar2002655

[29] M. C. Misuraca , E. Moulin , Y. Ruff , N. Giuseppone , New J. Chem. 2014, 38, 3336–3349.

[30] D. I. Rozkiewicz , B. J. Ravoo , D. N. Reinhoudt , Langmuir 2005, 21, 6337–6343. 15982040 10.1021/la050438i

[31] T. Chang , D. I. Rozkiewicz , B. J. Ravoo , E. W. Meijer , D. N. Reinhoudt , Nano Lett. 2007, 7, 978–980. 17373853 10.1021/nl070005v

[32] L. Tauk , A. P. Schröder , G. Decher , N. Giuseppone , Nat. Chem. 2009, 1, 649–656. 21378957 10.1038/nchem.400

[33a] N. Sakai , S. Matile , J. Am. Chem. Soc. 2011, 133, 18542–18545; 22026813 10.1021/ja207587x

[33b] H. Hayashi , A. Sobczuk , A. Bolag , N. Sakai , S. Matile , Chem. Sci. 2014, 5, 4610–4614;

[33c] K.-D. Zhang , S. Matile , Angew. Chem. Int. Ed. 2015, 54, 8980–8983; 10.1002/anie.201503033 26079103

[34a] S. Weigelt , C. Busse , C. Bombis , M. M. Knudsen , K. V. Gothelf , T. Strunskus , C. Wöll , M. Dahlbom , B. Hammer , E. Lægsgaard , F. Besenbacher , T. R. Linderoth , Angew. Chem. Int. Ed. 2007, 46, 9227–9230; 10.1002/anie.200702859 17968866

[34b] S. Weigelt , C. Busse , C. Bombis , M. M. Knudsen , K. V. Gothelf , E. Lægsgaard , F. Besenbacher , T. R. Linderoth , Angew. Chem. Int. Ed. 2008, 47, 4406–4410; 10.1002/anie.200705079 18442149

[34c] N. A. A. Zwaneveld , R. Pawlak , M. Abel , D. Catalin , D. Gigmes , D. Bertin , L. Porte , J. Am. Chem. Soc. 2008, 130, 6678–6679. 18444643 10.1021/ja800906f

[35] R. Tanoue , R. Higuchi , N. Enoki , Y. Miyasato , S. Uemura , N. Kimizuka , A. Z. Stieg , J. K. Gimzewski , M. Kunitake , ACS Nano 2011, 5, 3923–3929. 21480643 10.1021/nn200393q

[36] W. Dai , F. Shao , J. Szczerbiński , R. McCaffrey , R. Zenobi , Y. Jin , A. D. Schlüter , W. Zhang , Angew. Chem. Int. Ed. 2016, 55, 213–217. 10.1002/anie.201508473 26768822

[37] A. Ciesielski , M. El Garah , S. Haar , P. Kovaříček , J.-M. Lehn , P. Samorì , Nat. Chem. 2014, 6, 1017–1023. 25343608 10.1038/nchem.2057

[38a] W. Zhou , N. Yao , G. Yao , C. Deng , X. Zhang , P. Yang , Chem. Commun. 2008, 5577–5579; 10.1039/b808800d 18997957

[38b] L. Liang , Z. Liu , Chem. Commun. 2011, 47, 2255–2257; 10.1039/c0cc02540b 21258740

[38c] Z.-A. Lin , J.-N. Zheng , F. Lin , L. Zhang , Z. Cai , G.-N. Chen , J. Mater. Chem. 2011, 21, 518–524;

[38d] H. Wang , Z. Bie , C. Lü , Z. Liu , Chem. Sci. 2013, 4, 4298–4303.

[39] E. Aznar , M. Dolores Marcos , R. Martínez-Máñez , F. Sancenón , J. Soto , P. Amorós , C. Guillem , J. Am. Chem. Soc. 2009, 131, 6833–6843. 19402643 10.1021/ja810011p

[40] Y. Zhao , B. G. Trewyn , I. I. Slowing , V. S.-Y. Lin , J. Am. Chem. Soc. 2009, 131, 8398–8400. 19476380 10.1021/ja901831u

[41] K. C.-F. Leung , S. Xuan , C.-M. Lo , ACS Appl. Mater. Interfaces 2009, 1, 2005–2012. 20355826 10.1021/am900367a

[42] C.-H. Lee , S.-H. Cheng , I.-P. Huang , J. S. Souris , C.-S. Yang , C.-Y. Mou , L.-W. Lo , Angew. Chem. Int. Ed. 2010, 49, 8214–8219; 10.1002/anie.201002639 20865709

[43] D. P. Ferris , P. R. McGonigal , L. S. Witus , T. Kawaji , M. M. Algaradah , A. R. Alnajadah , M. S. Nassar , J. F. Stoddart , Org. Lett. 2015, 17, 2146–2149. 25894019 10.1021/acs.orglett.5b00740

[44a] A. G. Torres , M. J. Gait , Trends Biotechnol. 2012, 30, 185–190; 22260747 10.1016/j.tibtech.2011.12.002

[44b] G. Gasparini , G. Sargsyan , E.-K. Bang , N. Sakai , S. Matile , Angew. Chem. Int. Ed. 2015, 54, 7328–7331; 10.1002/anie.201502358 25925500

[45] F. M. Mansfeld , H. Y. Au-Yeung , J. K. M. Sanders , S. Otto , J. Syst. Chem. 2010, 1, 12.

[46] F. della Sala , E. R. Kay , Angew. Chem. Int. Ed. 2015, 54, 4187–4191; 10.1002/anie.201409602 PMC4409818 25973468

[47a] A. C. Templeton , M. P. Wuelfing , R. W. Murray , Acc. Chem. Res. 2000, 33, 27–36; 10639073 10.1021/ar9602664

[47b] X.-M. Li , J. Huskens , D. N. Reinhoudt , J. Mater. Chem. 2004, 14, 2954–2971.

[48] S. Borsley, E. R. Kay, *Chem. Commun* **2016**, DOI: 10.1039/C6CC00135A. 10.1039/c6cc00135a 27001937

[49] P. Nowak , V. Saggiomo , F. Salehian , M. Colomb-Delsuc , Y. Han , S. Otto , Angew. Chem. Int. Ed. 2015, 54, 4192–4197; 10.1002/anie.201409667 25663040

[50] Y. Han , P. Nowak , M. Colomb-Delsuc , M. P. Leal , S. Otto , Langmuir 2015, 31, 12658–12663. 26514180 10.1021/acs.langmuir.5b03673

[51] T. Sato , T. Ohishi , Y. Higaki , A. Takahara , H. Otsuka , Polym. J. 2016, 48, 147–155.

[52a] C. B. Murray , C. R. Kagan , M. G. Bawendi , Science 1995, 270, 1335–1338;

[52b] C. J. Kiely , J. Fink , M. Brust , D. Bethell , D. J. Schiffrin , Nature 1998, 396, 444–446;

[52c] B. L. V. Prasad , C. M. Sorensen , K. J. Klabunde , Chem. Soc. Rev. 2008, 37, 1871–1883; 18762836 10.1039/b712175j

[52d] C. R. Kagan , C. B. Murray , Nat. Nanotechnol. 2015, 10, 1013–1026. 26551016 10.1038/nnano.2015.247

[53] E. Auyeung , R. J. Macfarlane , C. H. J. Choi , J. I. Cutler , C. A. Mirkin , Adv. Mater. 2012, 24, 5181–5186. 22810947 10.1002/adma.201202069

[54a] A. M. Kalsin , M. Fialkowski , M. Paszewski , S. K. Smoukov , K. J. M. Bishop , B. A. Grzybowski , Science 2006, 312, 420–424; 16497885 10.1126/science.1125124

[54b] I. Hussain , Z. Wang , A. I. Cooper , M. Brust , Langmuir 2006, 22, 2938–2941; 16548536 10.1021/la053126o

[54c] R. Klajn , K. J. M. Bishop , M. Fialkowski , M. Paszewski , C. J. Campbell , T. P. Gray , B. A. Grzybowski , Science 2007, 316, 261–264; 17431176 10.1126/science.1139131

[54d] S. I. Lim , C.-J. Zhong , Acc. Chem. Res. 2009, 42, 798–808. 19378982 10.1021/ar8002688

[55] S. A. Berg , B. J. Ravoo , Soft Matter 2014, 10, 69–74. 24651686 10.1039/c3sm51515j

[56] W. Wei , L. Wu , C. Xu , J. Ren , X. Qu , Chem. Sci. 2013, 4, 1156–1162.

[57a] Y. Yi , H. Xu , L. Wang , W. Cao , X. Zhang , Chem. Eur. J. 2013, 19, 9506–9510; 23754765 10.1002/chem.201301446

[57b] H. Ying , Y. Zhang , J. Cheng , Nat. Commun. 2014, 5, 3218; 24492620 10.1038/ncomms4218 PMC4438999

[57c] S. Ji , W. Cao , Y. Yu , H. Xu , Angew. Chem. Int. Ed. 2014, 53, 6781–6785; 10.1002/anie.201403442 24842614

[57d] S. Billiet , K. De Bruycker , F. Driessen , H. Goossens , V. Van Speybroeck , J. M. Winne , F. E. Du Prez , Nat. Chem. 2014, 6, 815–821; 25143218 10.1038/nchem.2023

[57e] A. Sanchez-Sanchez , D. A. Fulton , J. A. Pomposo , Chem. Commun. 2014, 50, 1871–1874; 10.1039/c3cc48733d 24401844

[57f] B. Rasmussen , A. Sørensen , H. Gotfredsen , M. Pittelkow , Chem. Commun. 2014, 50, 3716–3718; 10.1039/c4cc00523f 24577496

[57g] R.-C. Brachvogel , M. von Delius , Chem. Sci. 2015, 6, 1399–1403; 10.1039/c4sc03528c PMC5811105 29560228

[57h] S. Gao , T. Stopka , M. Niggemann , Org. Lett. 2015, 17, 5080–5083; 26439888 10.1021/acs.orglett.5b02593

[57i] S. Kulchat , J.-M. Lehn , Chem. Asian J. 2015, 10, 2484–2496. 26213320 10.1002/asia.201500604

[58a] M. Mammen , S.-K. Choi , G. M. Whitesides , Angew. Chem. Int. Ed. 1998, 37, 2754–2794; 10.1002/(SICI)1521-3773(19981102)37:20<2754::AID-ANIE2754>3.0.CO;2-3 29711117

[58b] L. L. Kiessling , J. E. Gestwicki , L. E. Strong , Angew. Chem. Int. Ed. 2006, 45, 2348–2368; 10.1002/anie.200502794 PMC2842921 16557636

[59a] J.-M. Lehn , Angew. Chem. Int. Ed. 2013, 52, 2836–2850; 10.1002/anie.201208397 23420704

[59b] B. Grzybowski , S. Otto , D. Philp , Chem. Commun. 2014, 50, 14924–14925; 10.1039/c4cc90422b 25343613

[59c] J.-M. Lehn , Angew. Chem. Int. Ed. 2015, 54, 3276–3289; 10.1002/anie.201409399 25582911

[59d] E. Mattia , S. Otto , Nat. Nanotechnol. 2015, 10, 111–119. 25652169 10.1038/nnano.2014.337

[60a] L. Cusack , R. Rizza , A. Gorelov , D. Fitzmaurice , Angew. Chem. Int. Ed. Engl. 1997, 36, 848–851;

[60b] L. Cusack , S. N. Rao , D. Fitzmaurice , Chem. Eur. J. 1997, 3, 202–207. 24022948 10.1002/chem.19970030206

[61a] V. Saggiomo , U. Luening , Chem. Commun. 2009, 3711–3713; 10.1039/b902847a 19557257

[61b] V. Saggiomo , C. Goeschen , R. Herges , R. Quesada , U. Luening , Eur. J. Org. Chem. 2010, 2337–2343;

[61c] X. Wu , N. Busschaert , N. J. Wells , Y.-B. Jiang , P. A. Gale , J. Am. Chem. Soc. 2015, 137, 1476–1484. 25625547 10.1021/ja510063n

[62] M. J. Barrell , A. G. Campaña , M. von Delius , E. M. Geertsema , D. A. Leigh , Angew. Chem. Int. Ed. 2011, 50, 285–290; 10.1002/anie.201004779 20954231

